# Diagnostic à terme de jumeaux conjoints

**DOI:** 10.11604/pamj.2020.35.8.14062

**Published:** 2020-01-10

**Authors:** Imane Benchiba, Mohammed Karam Saoud, Nisrine Mamouni, Sanaa Errarhay, Chahrazad Bouchikhi, Banani Abdelaziz

**Affiliations:** 1 Service Gynécologie-Obstétrique I, CHU Hassan II de Fez, Maroc

**Keywords:** Jumeaux conjoints, malformations, diagnostic échographique anténatal, Conjoined twins, malformations, prenatal diagnostic ultrasound

## Abstract

Les jumeaux conjoints sont une malformation rare des grossesses gémellaires monozygotes et mono-amniotiques. Nous rapportons la découverte anténatale de jumeaux conjoints dicéphale à 37 SA. Le pronostic fœtal de cette malformation reste très sombre, nécessitant un recours à une interruption de la grossesse si le diagnostic est posé précocement. A partir de cette observation et une brève discussion, nous faisons un rappel sur cette pathologie rarissime, sur son diagnostic échographique essentiel pour établir une stratégie de prise en charge post natale. Son pronostic reste le plus souvent très réservé.

## Abstract (en)

Conjoined twins are a rare malformation of monozygotic and mono-amniotic twin pregnancies. We report the case of a patient in whom dicephalic conjoined twins was diagnosed in the antenatal period at 37 week amenorrhea. Fetal prognosis is very poor and, if the diagnosis has been made early, it most often requires therapeutic abortion. The purpose of this case study was to remind clinicians of this very rare disease, of the importance of ultrasound as an essential diagnostic tool to implement a strategy to manage patients in the postpartum period and of prognosis which is most often very guarded.

## Introduction

Les jumeaux conjoints sont une anomalie très rare concernant les grossesses gémellaires monozygotes. Ils sont dus à une division incomplète du disque embryonnaire au-delà du 13^ème^ jour gestationnel [1]. Leur classification se fait selon le site de connexions. Le diagnostic anténatal précoce de cette pathologie est nécessaire afin d'assurer un suivi adapté et une éventuelle décision obstétricale adéquate.

## Patient et observation

Il s'agit d'une patiente âgée de 24 ans, sans antécédents pathologiques notables, sans de notion de consanguinité, G1P0. Grossesse actuelle estimée à 37 SA selon une date des dernières règles précise suivie chez un gynécologue privé, ayant bénéficié d'une échographie à 17 SA n'ayant pas posé le diagnostic de jumeaux conjoints puis patiente perdue de vue. Consulte en urgence obstétricale en début de travail.

L'examen clinique à l'admission trouve une patiente consciente, normo tendu, normo carde, eupnéique et apyrétique. L'examen obstétrical trouve une hauteur utérine à 34cm, BCF positif dans un seul foyer à 150 battements/min, contractions utérines positives. Au spéculum col macroscopiquement normal, pas de métrorragie ni d'hydrorrhée. Au toucher vaginal, col dilaté à 1 doigt large, effacé à 60% central.

**Figure 1:**
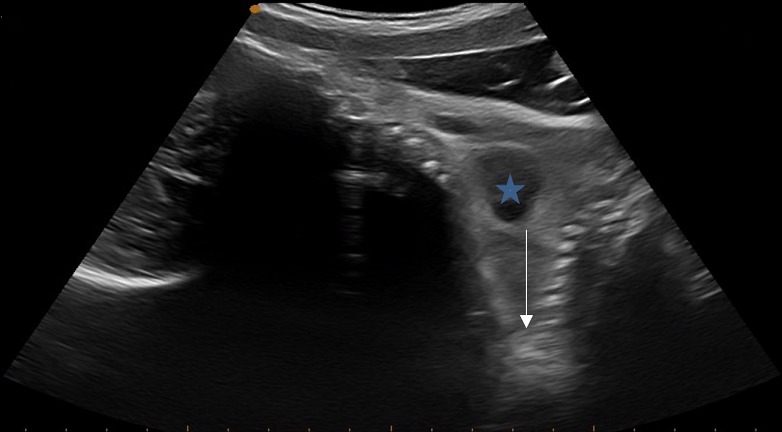
Image échographique montrant les deux rachis fusionnant (flèche bleue) et un cœur unique au milieu (étoile bleue)

**Figure 2:**
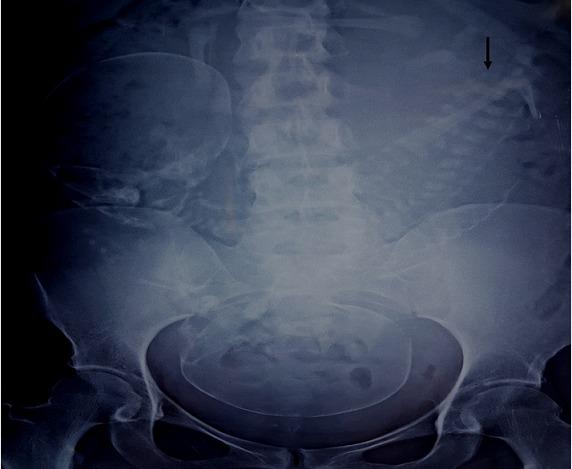
Image de la radiographie du contenu utérin montrant les deux têtes fœtales et les deux rachis fusionnant au niveau lombaire (flèche noire)

La patiente a bénéficié d'une échographie obstétricale (Figure 1) objectivant une grossesse gémellaire monochoriale monoamniotique avec fusion des deux rachis au niveau lombaire et la présence d'un cœur unique, le nombre des membres supérieurs et inférieurs est difficile à déterminer. L'aspect échographique fait évoquer le diagnostic de jumeaux conjoints. La patiente a bénéficié aussi d'une radiographie du contenu (Figure 2) montrant deux têtes fœtales avec deux rachis fusionnant au niveau de la région lombaire.

**Figure 3:**
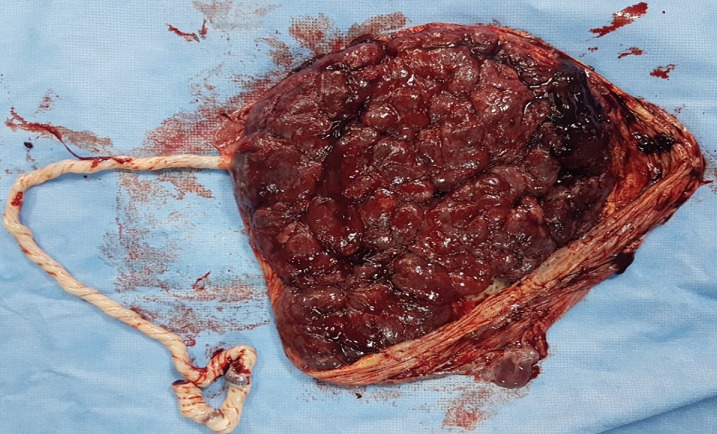
Placenta monochorial mono amniotique avec un cordon à quatre vaisseaux

**Figure 4:**
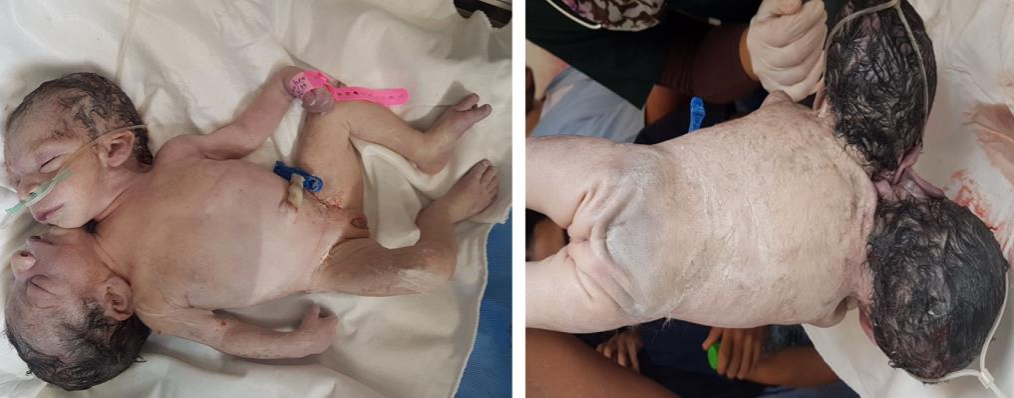
Image post natale des jumeaux conjoints vue antérieure et postérieure

Après avoir informé la patiente du pronostic de l'accouchement et du pronostic fœtal la décision était de la césariser. L'extraction était difficile vu que la 2^ème^ tête était en hyper extension. L'Apgar à la 1^ère^ minute était de 5 dixième devenant 7 dixième à la 5^ème^ minute. Le poids de naissance était de 3300g. L'examen du délivre (Figure 3) objective des anomalies vasculaires, trois artères et une veine. Les jumeaux (Figure 4) ont été examinés par les pédiatres dont l'examen initial a décelé une ectopie cardiaque avec deux trachées et deux œsophages, aucune autre malformation n'a pu être objectivée cliniquement. Les jumeaux ont présenté une détresse respiratoire intubée au niveau des deux trachées ventilées avec échec des mesures de réanimation, décédés à H3 de vie.

## Discussion

Les jumeaux conjoints sont dus d'un défaut de clivage des grossesses monozygotes au stade du disque embryonnaire. La première observation in utero par échographie de jumeaux conjoints [2] est due à Wilson en 1976. Le sexe féminin est plus fréquent avec un ratio de 1,6 à 3. Leur fréquence est estimée entre un pour 40000 à un pour 80 000 naissances, mais seulement à un pour 200 000 enfants nés vivants. Il n'y a pas de cas connu de triplets ou de quadruplets conjoints [2]. Plusieurs classifications ont été décrites en fonction du site d'union, des organes communs et de la symétrie. Saint-Hilaire en a établi une en 1832, se basant sur la description du site d'union externe et sur la symétrie. Il en résulte huit types de duplication complète: les jumeaux conjoints céphalopages, thoracopages, omphalopages, ischiopages, parapages, craniopages, pygopages et rachipages [3]. Le cas que nous rapportons est un cas rare résultant d'une duplication incomplète des jumeaux appelée dicéphales (un seul corps et 2 têtes).

De nombreux cas rapportés dans la littérature internationale sont diagnostiqués à partir de la 9^ème^ semaine d'aménorrhée. En France, compte tenu de la pratique des échographies au cours de la grossesse, le diagnostic de jumeaux conjoints devrait pouvoir presque toujours être fait lors de l'examen du premier trimestre, entre la 10^ème^ et la 13^ème^ semaine d'aménorrhée. Certaines formes incomplètes peuvent être difficiles à diagnostiquer; elles devront être élucidées lors de la deuxième échographie réalisée autour de 22 semaines d'aménorrhée. L'analyse ne doit pas s'arrêter au diagnostic de jumeaux conjoints (JC), mais doit aussi rechercher des anomalies malformatives associées compte tenu de leur plus grande fréquence et de l'aggravation du pronostic que leur découverte va entrainer. Les autres techniques d'imagerie (radio du contenu utérin et imagerie par résonance magnétique (IRM)) peuvent parfois apporter un complément d'information, à visée pronostique, mais ceci tardivement. L'embryoscopie est à la disposition de peu d'équipes. Dans notre cas, la patiente avait bénéficié d'une échographie a 14 SA d'aménorrhée qui malheureusement n'a pas pu poser le diagnostic de jumeaux conjoint due probablement à un opérateur inexpérimenté. Dans le cas où le diagnostic a été posé tardivement au 2^ème^ trimestre ou au 3^ème^ trimestre, l'intérêt de la surveillance à ce stade se voit dans le cas où la décision de conserver la grossesse a été prise afin de rechercher l'apparition d'éventuelles anomalies associées passées inaperçues jusque-là, et de de donner des précisions utiles à l'équipe chirurgicale pour mieux poser la stratégie postnatale.

Le pronostic des jumeaux conjoints reste très réservé. Pour Romero, 39% des jumeaux conjoints sont mort-nés et 34% meurent dans les heures qui suivent la naissance [4]. La survie des jumeaux dépend du type d'union (organes en commun) et des autres anomalies associées. Actuellement, seules quelques équipes chirurgicales pédiatriques sont capables d'envisager une chirurgie de séparation des deux jumeaux, à condition qu'il n'y ait pas d'association malformative sévère. Quand l'échographie anténatale montre l'existence de malformations sévères une interruption médicale de grossesse doit être proposée après avis demandé auprès d'une équipe chirurgicale compétente.

## Conclusion

Le pronostic des JC dépend essentiellement du site et de l'extension des organes fusionnés. La première échographie à 12 ou 13 SA sera importante pour rechercher ce diagnostic et pour évaluer les éventuelles anomalies associées. Le diagnostic précoce et exact de JC reste un défi, mais est important pour planifier un accueil de l'enfant en cas de malformation curable. Si celle-ci est incurable, une interruption médicale de la grossesse précoce sera possible, évitant un accouchement tardif et traumatisant aussi bien sur le plan physique que psychologique [5].

## Conflits des intérêts

Les auteurs ne déclarent aucun conflit d’intérêts.
